# Effect of spatial resolution on cluster detection: a simulation study

**DOI:** 10.1186/1476-072X-6-52

**Published:** 2007-11-27

**Authors:** Al Ozonoff, Caroline Jeffery, Justin Manjourides, Laura Forsberg White, Marcello Pagano

**Affiliations:** 1 Department of Biostatistics, Boston University School of Public Health, 715 Albany Street, Boston, MA 02118, USA; 2 Department of Biostatistics, Harvard School of Public Health, 655 Huntington Avenue, Boston, MA 02115, USA

## Abstract

**Background:**

Aggregation of spatial data is intended to protect privacy, but some effects of aggregation on spatial methods have not yet been quantified.

**Methods:**

We generated 3,000 spatial data sets and evaluated power of detection at 12 different levels of aggregation using the spatial scan statistic implemented in SaTScan v6.0.

**Results:**

Power to detect clusters decreased from nearly 100% when using exact locations to roughly 40% at the coarsest level of spatial resolution.

**Conclusion:**

Aggregation has the potential for obfuscation.

## 1 Introduction

The Centers for Disease Control and Prevention (CDC) define surveillance to be the ongoing, systematic collection, analysis, interpretation, and dissemination of data about a health-related event for use in public health action to reduce morbidity and mortality and to improve health [1]. To control and prevent disease, it is surely important to be vigilant for infectious disease outbreaks or geographic areas of notably high chronic disease incidence. Indeed this is a primary aim of public health surveillance, and explains in part why surveillance plays an integral role in public health practice [2].

When caring for a single patient, the clinician understandably desires as much diagnostic information as possible, and at the highest possible level of precision. Analogously, a public health professional is concerned with diagnosing a public ailment, and should similarly desire all available information with the greatest possible level of precision. Thus it is noteworthy, in the context of public health surveillance, that for reasons of privacy, information is sometimes destroyed or intentionally degraded before being proffered to the analyst.

The argument to protect patient data for reasons of privacy could also be used to shield these data from clinicians. In a clinical setting, we choose not to protect the privacy of the patient by hiding relevant information from the clinician, because it is patently silly to do so. However, we often suffer from a similarly framed argument to obscure population level data, even when addressing matters of concern to the public health.

We argue that one important reason to retain important, specific information such as precise location is that the "requisite" aggregation for privacy necessarily reduces the power available for outbreak detection. To balance the cost of this and other troubles for spatial analysis [3], aggregation does indeed make it more difficult to identify individual patients. This is crucial if the data are made publicly available or if there are other reasons to safeguard privacy, but it also makes an already challenging surveillance task even more difficult.

A growing body of literature addresses statistical protection of privacy and its effects on analysis of surveillance data. Cox has written a useful survey of the general problem of confidentiality within small geographic areas, and the impacts of privacy concerns on public health policy and practice [4].

Armstrong et al. thoroughly discuss the design and implementation of several different approaches to protect privacy in the context of spatial analyses [5]. Importantly, methods were evaluated both on the impact on analysis as well as the effectiveness of preserving confidentiality. Yet the restriction of the quantitative assessment to the Cuzick-Edwards test statistic [6], which is no longer commonly used for spatial surveillance [7,8], limits the application of this knowledge to a surveillance setting. Further, data with exact locations were not considered for this evaluation.

Waller and colleagues have written extensively on factors that may influence power of cluster detection methods. For example, they have studied the effects of geographic scale on focused tests of clustering [9,10], and the importance of cluster location amidst a heterogeneous underlying population [11]. Notably, this group has investigated more than one statistical method, using several different measures for evaluation. However these studies generally use focused tests of clustering, where a putative exposure source has been identified *a priori*, whereas surveillance purposes typically require a general test of clustering [12].

Just as we trust clinicians and hospital personnel with sensitive and confidential information, so too, one can argue, we should find trustworthy individuals to handle surveillance data responsibly.

Informatics-based approaches offer a potential compromise to the trade-off between privacy and surveillance utility. For example, development of automated surveillance algorithms might allow sensitive data to be analyzed without human intervention [13]. But in order to evaluate the benefit that such an approach might provide, we must first better understand the costs in performance that the obfuscation or destruction of information may cause.

We reported briefly [14] that there is an undesirable loss of power to detect disease outbreaks when the spatial information provided is degraded from a continuous scale of measurement to a coarser, aggregate level. For example, often only a patient's ZIP code is available to a surveillance system, instead of the patient's listed residential address. Similar results have appeared in contemporaneous work [15], and a recent paper by the same group further confirms this basic premise [16]. However, those studies focused solely on exact locations compared to a single level of aggregation.

In our present work, we add to these previous results by considering multiple levels of aggregation. Using synthetic data, we systematically quantify the loss of cluster detection performance as a function of spatial resolution, while limiting confounding influences from a variety of complex factors that affect spatial analyses. We may interpret these results relative to geographic scales we might encounter while surveilling a large metropolitan city. In this way, we attempt to clarify the price one pays for aggregation, and in turn to better inform future policy decision-makers.

## 2 Methods

### 2.1 Data

**Figure 1:**
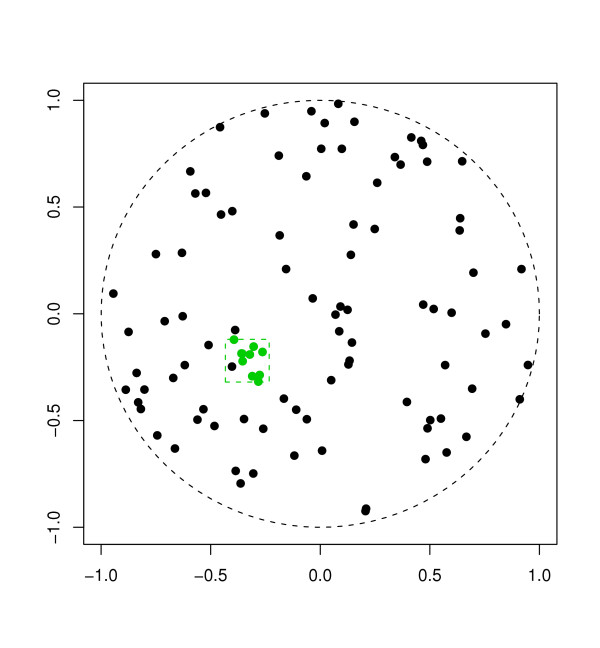
**Illustration of a simulated cluster**. 90 points were distributed uniformly on the unit circle, and 10 additional "outbreak" points form the square "cluster" left of center.

We designed a simulation study to determine the effect of spatial aggregation on power to detect spatial clusters. Random samples of size 90 were drawn from an underlying uniform distribution on the unit disk (i.e. the Euclidean circle of radius one). Atop this background sample, we then superimpose a simulated cluster consisting of 10 points uniformly distributed in a small square at a location randomly determined for each simulated data set (Figure 1). Thus each simulated data set consists of a total sample of 100 points. Although the clusters are not defined by circles, for ease of discussion we speak of a cluster "radius" to mean the radius of the circle inscribed within the square cluster boundary. In the occasional instance where the cluster center falls within one radius of the unit disk boundary, we require that all 10 cluster points lay within the intersection of the cluster boundary and the unit disk.

We generated three separate sets of simulated data with cluster radii of 0.025, 0.05 and 0.10, corresponding to disease clusters with a geographical extent equal to 2.5%, 5%, or 10% respectively of the radius of the study area. Although this results in clusters of different intensities, the corresponding relative risks are quite large (greater than 10) for all simulations. For each cluster radius, we generate 1, 000 data sets under these conditions, or a total of 3, 000 data sets for the entire simulation study.

**Figure 2:**
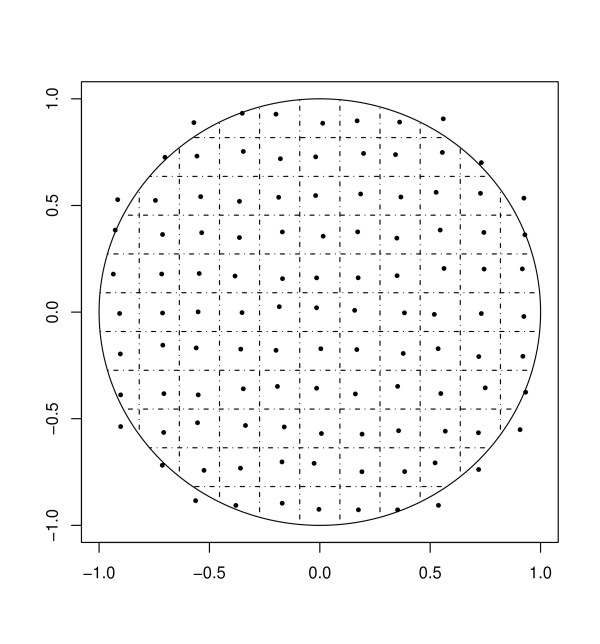
**Illustration of spatial aggregation**. One of 12 levels of spatial aggregation used in this study. Grid lines define spatial regions of aggregation, and representative points are chosen randomly within each region. All simulated points are reassigned to the representative point of the appropriate region.

To simulate spatial aggregation at different geographic scales, we use a sequence of 12 uniform grids of varying spacing, superimposed on the unit disk. The levels of aggregation are chosen according to their corresponding grid spacing, ranging from 15 grid squares per side (length of grid square 0.067) to four grid squares per side (length of grid square 0.25). Throughout, we use the average distance between grid points (equivalently, the average diameter of an aggregation region) as an index of the level of spatial aggregation (Figure 2).

By assigning all simulated data points to the nearest grid point, these grids thereby define spatial regions of aggregation. Prior to analysis, we modified each grid by adding small amounts of bivariate jitter to each grid point (i.e. region center). Our purpose was to mitigate the high degree of spatial regularity across a uniform grid of assignment points, and in part to reflect the non-uniform nature of administrative regions as they appear in real systems. We note however that the use of a uniform population distribution implies constant population densities across administrative region, something unlikely to be seen in a real system.

### 2.2 Statistical analysis

We use SaTScan version 6.0 (2005) with a purely spatial Bernoulli model, with cluster size constrained to be no greater than 25% of the population. Statistical significance of spatial clusters is determined using a nominal Type I error rate of 0.05.

Our primary outcome is the proportion of simulated data sets, under each level of aggregation, for which SaTScan accurately detects the simulated cluster. We denote this proportion as the power to detect clusters. In order to ensure that the cluster detected by SaTScan is sufficiently close in space to the true cluster location, we record a detection as successful if and only if the identified cluster center is within one cluster radius of the true cluster center. We also record the proportion of false detections, defined as any cluster identification with center more than one cluster radius from the true cluster center, or failure of any identified cluster to achieve significance level (i.e. p-value) below 0.05.

To measure the spatial accuracy of cluster detection, we further consider the identification (correctly or not) of individual data points in a significant disease cluster. Within each simulated data set, there were 10 points of 100 that comprised the simulated cluster. For these "cluster points", we calculate the proportion correctly included in a SaTScan-identified cluster with p-value below 0.05. Similarly for the remaining 90 "non-cluster points", we calculate the proportion incorrectly included in a statistically significant SaTScan-identified cluster. These proportions are analogous to traditional definitions of sensitivity and 1 minus specificity, respectively, where we compare the classification via SaTScan of points involved in a cluster to the "gold standard" of cluster status as determined by simulation design.

## 3 Results

**Figure 3:**
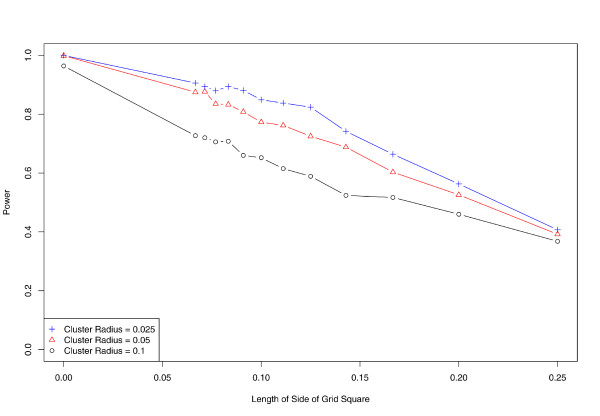
**Effect of aggregation on power**. As spatial data are aggregated, power to detect clusters decreases. Horizontal axis denotes level of spatial aggregation, determined by radius of aggregation region; vertical axis denotes proportion of simulated clusters correctly identified at significance level *α *= 0.05.

Figures 3 through 6 illustrates our results. For all three sets of simulations, power decreases as the size of aggregation regions increases. These simulated clusters are sufficiently large so that the power to detect for all three cluster radii is nearly 100% when exact locations are used; this decreases to roughly 40% at the coarsest level of aggregation, which corresponds to a more than halving of the probability of successful detection (Figure 3).

**Figure 4:**
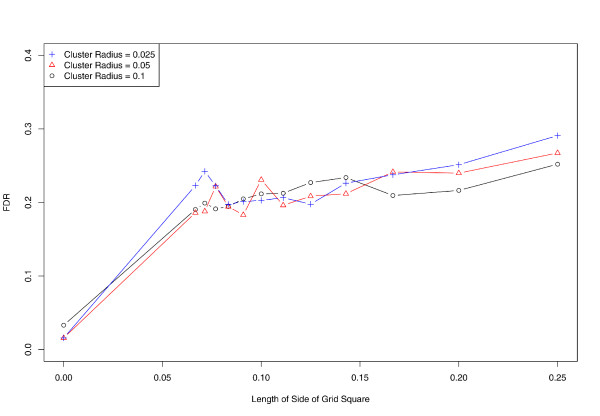
**Effect of aggregation on false detection rate**. Vertical axis denotes proportion of simulations where spurious clusters are detected.

**Figure 5:**
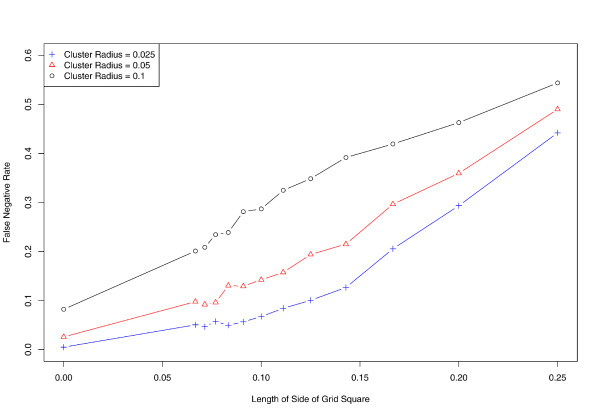
**Effect of aggregation on sensitivity**. Identification of cases involved in an outbreak becomes more difficult as data are aggregated. Vertical axis denotes proportion of cases falsely identified as outside the disease cluster (false negatives).

**Figure 6:**
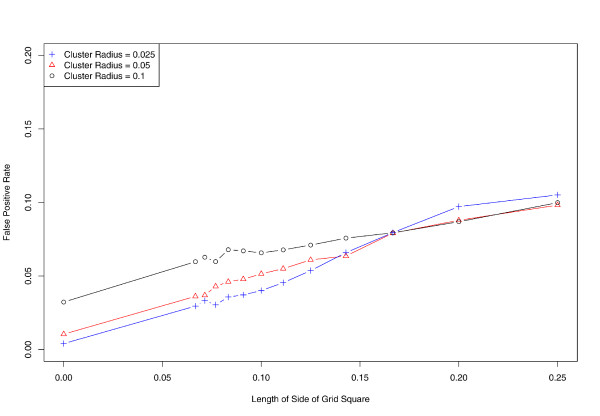
**Effect of aggregation on specificity**. Vertical axis denotes proportion of cases falsely identified as inside the cluster (false positives).

Using exact locations, the false detection rate is approximately 2%. In the presence of any level of aggregation, the false detection rate increases to nearly 20% or higher in all of our simulations (Figure 4). This rate appears to increase slowly for greater levels of aggregation.

We further evaluate the effect of aggregation on the sensitivity and specificity of SaTScan (Figures 5 and 6). While performance is nearly ideal when using exact locations, the proportion of false negatives rises to almost 50% at the coarsest level of aggregation. In concordance with our earlier results, sensitivity tends to decrease as spatial aggregation increases, while the false positive fraction (1 minus specificity) follows an inverse and nearly monotonic association.

## 4 Discussion

Our results are noteworthy for a number of reasons. First, we have used more than two levels of aggregation in an effort to estimate the incremental effect of this aggregation on the power of cluster detection. Second, we have further investigated the effect of aggregation on the rate of false detection. Finally, when viewed in the context of similar studies, our results add to a body of evidence that the underlying relationships reported appear robust to differing geographies and population distributions.

Our calculation of power and false detection differs from the same measures as otherwise used in an important way. We expect a certain proportion of spurious "clusters" to arise by chance alone. Thus we have placed an additional requirement on what we denote a successful identification of a cluster, namely that the identified cluster be proximal to the true cluster as determined by the simulation design. Because our simulations involve only one cluster per data set, an identification far from the true cluster is genuinely spurious and must be considered a false detection in this context. Indeed, for practical purposes such an identification might divert resources for investigation to a geographic area not related to the true outbreak or cluster present in the data.

To place our results in context, consider the metropolitan Boston area. The city and adjacent suburbs can be enclosed in a circle of radius roughly 7, 500 meters. Although the size of city ZIP codes and census tracts varies, an approximate median radius for Boston ZIP codes is roughly 1, 500 meters, or 20% of the region radius. Boston census tracts have an approximate median radius of 500 meters, or 6.7% of the region radius. Thus census tract and ZIP code aggregation of Boston data corresponds roughly to our first and penultimate levels of aggregation respectively. Likewise, the simulated clusters of radii 0.025, 0.05, and 0.10 correspond to disease outbreaks smaller than one census tract, about one census tract, or several census tracts (perhaps a small ZIP code) respectively.

The number of false detections rose well above the nominal alpha level when spatial data were aggregated. Interestingly, the level of aggregation does not appear to be a major contributor to false alarms; rather, there is an immediate increase upon aggregation above the nominal false alarm rate, with little additional increase for further aggregation. To our knowledge, this has not been reported previously. Since false alarms form a major limitation to the actionable consequences of cluster detection, this issue should be considered carefully. Even in situations where loss of power is not severe, the increase in false detection rates may impose further limits of the utility of spatial methods when using aggregated data.

Our study is limited in several ways. We have only included an evaluation of SaTScan as a test of clustering, although we have seen similar results using other methods [14]. The use of synthetic data is both helpful and harmful to generalizability of results. There are few populations that even approximate a homogeneous and uniform distribution, and thus the simulated data sets do not reflect a realistic surveillance scenario. However, using a homogeneous distribution removes some of the potentially confounding interactions between cluster location, geography, population distribution, and spatial methods. Thus despite its limitations, our study contributes to an understanding of the complex association between spatial resolution and power of detection.

We chose not to investigate spatio-temporal methods (implemented for example with a space-time scan, also available using SaTScan). Space-time interactions imply greater complexity when considering effects of spatial aggregation (or indeed, temporal aggregation), and the potential parameter space of simulation studies increases greatly as well. For this and other reasons, the effect of spatial aggregation (or indeed, temporal aggregation) in a cluster detection context remains an area for further investigation.

## Competing interests

The author(s) declare that they have no competing interests.

## Authors' contributions

AO and MP conceived of the study, participated in the design, and drafted the manuscript. AO, CJ, and JM were responsible for statistical programming and data analysis. All authors read and approved the final manuscripts.
